# Complex Regulation of the Pericellular Proteolytic Microenvironment during Tumor Progression and Wound Repair: Functional Interactions between the Serine Protease and Matrix Metalloproteinase Cascades

**DOI:** 10.1155/2012/454368

**Published:** 2012-02-20

**Authors:** Cynthia E. Wilkins-Port, Stephen P. Higgins, Craig E. Higgins, Issey Kobori-Hotchkiss, Paul J. Higgins

**Affiliations:** Center for Cell Biology and Cancer Research, Albany Medical College, 47 New Scotland Avenue, Albany, NY 12208, USA

## Abstract

Spatial and temporal regulation of the pericellular proteolytic environment by local growth factors, such as EGF and TGF-*β*, initiates a wide repertoire of cellular responses coupled to a plasmin/matrix metalloproteinase (MMP) dependent stromal-remodeling axis. Cell motility and invasion, tumor metastasis, wound healing, and organ fibrosis, for example, represent diverse events controlled by expression of a subset of genes that encode various classes of tissue remodeling proteins. These include members of the serine protease and MMP families that functionally constitute a complex system of interacting protease cascades and titrated by their respective inhibitors. Several structural components of the extracellular matrix are upregulated by TGF-*β* as are matrix-active proteases (e.g., urokinase (uPA), plasmin, MMP-1, -3, -9, -10, -11, -13, -14). Stringent controls on serine protease/MMP expression and their topographic activity are essential for maintaining tissue homeostasis. Targeting individual elements in this highly interactive network may lead to novel therapeutic approaches for the treatment of cancer, fibrotic diseases, and chronic wounds.

## 1. Introduction

 Epithelial transdifferentiation or cellular “plasticity” refers to a specialized morphogenetic switch typified by loss of normal epithelial properties, a gain in the expression of genes generally restricted to the mesenchymal lineage and conversion of sessile, nonmotile cells to a migratory phenotype [1, 2]. While essential during development and organogenesis (i.e., embryonic patterning) (also termed Type 1 transition), this process is relatively limited in the adult organism, occurring during wound healing and regenerative repair [3–5] or, more atypically, in tissue fibrosis (Type 2) and tumor metastasis (Type 3) [6–9]. Whether a true epithelial-mesenchymal-myofibroblast transition, or a more intermediate state of transdifferentiation, contributes to the pathophysiology of human fibrotic disease, however, is the subject of considerable debate [10–16].

 The temporal and spatial regulation of cellular plasticity, as well as the subsequent restitution of an epitheloid phenotype, is likely a collective response to specific growth factors (individually or in combination) and informational cues from the extracellular environment [2, 8, 17]. The nature of the initiating stimulus as well as the underlying pathology and associated genetic reprogramming also impact temporal control versus persistence of the plastic restructuring. Epidermal growth factor receptor (EGFR) amplification and alterations in the transforming growth factor-*β* (TGF-*β*) signal transduction network, for example, frequently accompany epithelial tumor progression from a benign or noninvasive lesion to an aggressive, metastatic carcinoma [18–20]. During this transition, and despite increased autocrine/paracrine expression of TGF-*β*, cells often become refractory to the normally growth-suppressive effects of TGF-*β* family members due, in part, to downregulation of TGF-*β* receptors and/or anomalies in TGF-*β*-initiated signaling pathways [20, 21]. Similarly, in tissue fibrotic disorders, differentiation, proliferation, and subsequent interstitial accumulation of “fibroblastoid” elements in the kidney and lung (two of the most well-studied organ systems in the context of fibroproliferative disease) appear to result from a programmed, but persistent, response to several profibrotic cytokines, the most prominent of which is TGF-*β* [11].

## 2. The Serine Protease-Matrix Metalloproteinase Cascade in Tissue Remodeling

 TGF-*β* promotes cellular motile and invasive properties, as well as the emergence of the plastic cohort, through expression of a subset of genes that encode various classes of stromal remodeling proteins [22, 23]. These include members of the serine protease and matrix metalloproteinase (MMP) families and their respective inhibitors which, paradoxically, support matrix disruptive as well as stabilizing processes. Several structural components of the extracellular matrix [24, 25] are, in fact, upregulated by TGF-*β* as are matrix-active proteases (e.g., urokinase (uPA), plasmin, MMP-1, -3, -9, -10, -11, and -13) and protease inhibitors [26–29]. Stringent controls on serine protease/MMP transcription, duration of expression, and topographic activity are essential for maintaining tissue homeostasis in the intact organism as well as in organotypic systems [30]. Proteolytic networks within the pericellular microenvironment, moreover, are frequently activated by the conversion of plasminogen to plasmin, a broad-spectrum protease. Plasmin, in turn, targets stromal elements directly while also activating several MMPs triggering a complex cascade leading to matrix degradation [31]. Upstream plasmin generation substantially impacts MMP-dependent stromal remodeling and, thereby, cellular invasive traits. Such *in vivo *pathologies can be elegantly modeled *in vitro *upon addition of EGF + TGF-*β*1 to malignant human epithelial cells to mimic the frequently observed TGF-*β*1 elevation in the tumor microenvironment and amplified EGFR signaling characteristic of late-stage cancers [32]. Combined EGF + TGF-*β*1 costimulation resulted in the synergistic upregulation of a defined set of proinvasive genes, the most prominent of which encodes plasminogen activator inhibitor-type-1 (PAI-1 or SERPINE1, the clade E member 1 of the family of serine protease inhibitors). PAI-1 is a potent and fast-acting inhibitor of uPA-dependent plasmin production [22, 23]. This finding is of considerable translational relevance since increased PAI-1 levels often occur in concert with epithelial cell plasticity, paralleling the requirement for enhanced cell motility [33]. The ability of TGF-*β* and/or EGF to increase PAI-1 expression in several cell types [26, 34] provides a potential mechanism for upstream titration of the MMP cascade via controlled generation of pericellular plasmin consequently modulating, both in time and space, extracellular matrix proteolysis and stromal remodeling. Indeed, elevated PAI-1 levels commonly accompany the development of such diverse pathologies as tumor progression, inflammation, hypertrophic scarring, atherosclerosis, thrombosis, myocardial infarction, diabetes, and the obesity-associated metabolic syndrome [11, 31, 35–40].

## 3. Focal Proteolysis: Regulation of Cell Migration and Signaling

**Figure 1:**
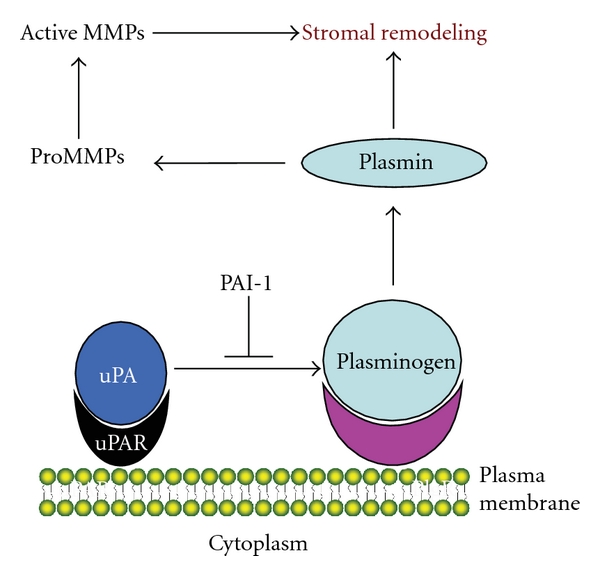
The plasmin/MMP axis in pericellular proteolytic control. uPA, tethered to its receptor (uPAR), converts plasminogen receptor- (PlgR-) bound plasminogen to the broad-spectrum protease plasmin that, in turn, activates several MMP family members. Collectively, plasmin and MMPs regulate the extent, duration, and locale of stromal remodeling.

 The contribution of PAI-1 as a promoting element in various disease states is thought to occur through multiple avenues involving proteolytic control, an essential aspect in the maintenance of a “stromal scaffold” that impacts cell survival, growth and transdifferentiation, cellular motile processes, and signal transduction. Focal proteolysis within the pericellular microenvironment is controlled primarily through mechanisms that regulate plasminogen activation at the cell surface that, in turn, affect MMP activation downstream with subsequent engagement of a complex tissue remodeling program [41] (Figure 1).

**Figure 2:**
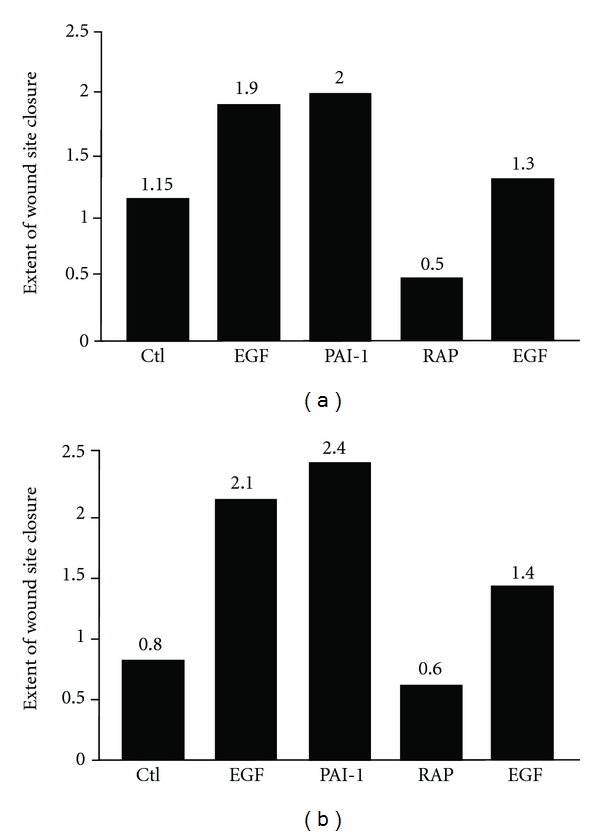
HaCaT II-4 cultures were grown to confluency, monolayers washed 2X with PBS and incubated in serum-free DMEM for 24 hours. Cultures were scrape-wounded with a P1000 pipette tip using a constant-pressure press, washed 3X to remove liberated cells, and returned to serum-free medium without (control) or with the following additives: EGF (10 ng/mL), PAI-1 (40 mM), RAP (5 *μ*g/mL), or the combination RAP (5 *μ*g/mL) + EGF (10 ng/mL). Initial wound sizes were measured with a calibrated ocular grid at multiple marked sites; 24 hours later, the injury sites were remeasured at the identical marked regions used to calibrate the initial denuded area. The extent of wound site closure (motility index = grid distance migrated) was plotted on the *y*-axis for each condition; shown are data from 2 representative experiments. PAI-1 stimulated cell migration to the same extent as EGF. Ctl: control unstimulated cultures.

Importantly, focalized proteolysis promotes the discrete release of several physiologically significant bioactive fragments and growth factors from the stromal compartment that influence cell proliferation and cell migration. MMP-dependent generation of degradation products of extracellular matrix structural elements, for example, affects both angiogenic and antiangiogenic activities with an impact on endothelial motile characteristics under *in vivo*-relevant conditions [42]. MMP-2 and MMP-9 cleave collagen IV, exposing cryptic epitopes that stimulate angiogenesis [43, 44] while matrikines such as arrestin, canstatin, tumstatin and metastatin, also generated from collagen IV, are antiangiogenic [41, 42]. Proteolytically derived fragments of collagen XVIII (endostatin and neostatin), collagen VIII (vastatin), collagen XV (restin), and perlecan (endorepellin) similarly exhibit anti-angiogenic properties [41, 42]. These fragments often compete with intact extracellular matrix molecules for binding to various cell surface receptors as one mechanistic basis for their effects [41]. A particularly important event involves the MMP-dependent release of laminin-332 fragments that promote epithelial cell migration. Indeed, the recombinant domain III of the laminin-332 *γ*2 chain (processed from laminin-332 by MT1-MMP, a membrane type 1 MMP, and MMP-2) binds to EGFR and initiates signaling events which culminate in enhanced cell motility [45–47]. Similarly, MMP-based proteolysis of fibronectin yields fragments that affect migration (MSF) [41, 42, 48], angiogenesis (anastellin) [49, 50], cell proliferation, and differentiation [41]. Stromal PAI-1 is itself a substrate for several extracellular proteases including elastase, MMP-3 and plasmin resulting in the generation of rather specific PAI-1 cleavage products [32, 33, 51–53]. Such “cleaved” PAI-1 is unable to bind its target plasminogen activators uPA and tissue-type PA (tPA) to inhibit plasmin-based proteolysis but retains the ability to bind to the low-density lipoprotein receptor-related protein-1 (LRP1) where it effectively augments cell migration through a uPA/tPA complex-independent interaction [54]. The mechanistic basis for this response is not clear but appears to involve LRP1 function as a key signaling mediator in several intracellular pathways as a consequence of, in part, interactions with multiple adaptor and scaffolding proteins [55]. LRP1 ligand binding and/or complex formation with additional surface receptors also activates specific mitogen-activated protein (MAP) and *src* kinases [56–60] stimulating cell proliferation [58, 61–63] and migration [54, 56, 64] with the motile outcome dependent on Rho family GTPases [64]. Alternatively, PAI-1 can also initiate signaling events that impact cell migration through engagement of LRP1 and the related very low-density lipoprotein receptor [65]. Indeed, different conformations of PAI-1 (active, latent as well as plasmin- or MMP-cleaved) all interact with LRP1 to enhance cell migration into physiological scaffolds or stromal equivalents [66]. The three forms of PAI-1 appear to increase LRP1-dependent cell motility via specific engagement of the Jak/Stat1 signaling pathway [54, 67, 68]. These data are consistent with recent findings that the migratory response to TGF-*β*1 + EGF in transformed human epidermal keratinocytes is PAI-1-dependent [22, 23] and that stabilized (i.e., long half-life) recombinant PAI-1 alone, in the absence of added growth factors, stimulates motility comparable to that attained by growth factor supplementation (Figure 2). The receptor-associated protein (RAP), an LRP1 antagonist which binds LRP1 and blocks interactions with all known ligands including PAI-1, inhibits the migration-promoting effects of PAI-1 in chemotactic and wound healing assays [54] and effectively inhibited EGF-stimulated motility, which is known to be dependent on LRP1/PAI-1 (Figure 2). While active PAI-1 is routinely cleared from the extracellular environment in a complex with uPA/uPAR/LRP1, latent and cleaved species of PAI-1, with a preserved motile function, remain embedded in the matrix likely serving as a reservoir to maintain cell movement [33]. Collectively, these data illustrate that serine protease/MMP-initiated proteolytic processing of the extracellular environment impacts multiple aspects with regard to the regulation of cell motility.

## 4. Tumor Microenvironment and Cutaneous Wound Repair: Sites of Interacting Proteolytic Cascades

**Figure 3:**
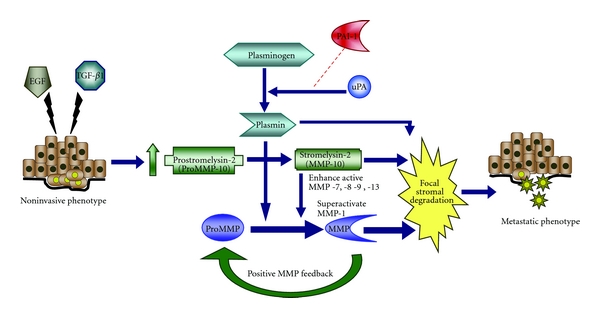
Model of TGF-*β*1 + EGF-enhanced plasmin-dependent collagen matrix remodeling and development of an invasive phenotype. In the presence of plasmin, increased MMP-10 levels promote MMP activation creating a proteolytic axis that accelerates collagen degradation through “superactivation” of MMP-1. STAT3 may act as a positive switch in this process, via promotion of EGF-stimulated proMMP-10 expression [23]. Upregulation of PAI-1 in response to TGF-*β*1 + EGF may subsequently shift this proteolytic balance, enabling PAI-1 to “titrate” the extent and locale of collagen matrix remodeling to facilitate stromal invasion. Indeed, PAI-1 induction occurs early in this transition and required for stimulated migration and collagen invasion since PAI-1 knockdown (with siRNA constructs) effectively inhibited both events [22].

Amplified MMP expression correlates with tumor aggressiveness, metastasis, and poor prognosis [69]. Not surprisingly, therefore, recent studies implicate several MMPs, including MMP-3, -7, -9, and -28 in triggering plasticity-related processes [69]. The combination of TGF-*β* + EGF effectively promotes epithelial-to-mesenchymal transition and upregulates MMPs-1, -3, -9, -10, and 14 [22, 23, 70, 71], coupling cellular invasive potential to a plasmin/MMP-10/MMP-1-dependent collagen-remodeling axis. As a proof-of-concept, an acute collagenolytic phenotype, linked to plasmin-dependent activation of stromelysin-2 (MMP-10), accompanies costimulation of malignant (HaCaT II-4) keratinocytes with TGF-*β*1 and EGF coincident with collagen invasion [32] (Figure 3). MMP-10, which is generally limited to epithelial cells [29, 72], targets proMMPs-1, -7, -8, -9, and -13, as well as collagens type III, IV, and V, gelatin elastin, fibronectin, proteoglycans, and laminin [30, 31]. MMP-10 induction in response to TGF-*β* + EGF suggests that precise control over its levels and activation are likely critical for cutaneous homeostasis. MMP-10, in fact, is not evident in intact skin but expressed during cutaneous injury repair localizing to migrating keratinocytes at the wound edge, suggesting a role in invasive behavior [73].

 Similar to other systems [29, 71, 72, 74], MMP-10 and MMP-1 are upregulated in TGF-*β*1 and/or EGF-stimulated human malignant keratinocytes maintained on collagen substrates [32]. The proenzyme forms of MMP-1 and MMP-10 are plasmin substrates. Following MMP-10 inhibition, however, the residual level of active plasmin-generated MMP-1 appears insufficient to initiate collagen dissolution [32]. This reflects the established ability of MMP-10 to “superactivate” or enhance MMP-1-dependent proteolysis, as well as that of MMP-8 and -13, and significantly enhances collagenolytic activity over that observed with plasmin alone [72, 75]. Although plasmin is clearly an important primary activator of this complex cascade, the cathepsins, like MMPs, also associate with tumor cell invasion. This is particularly true for the cysteine proteinases cathepsin L and cathepsin B, which degrade type-1 collagen and mobilize several MMPs (including MMP-1), respectively [76]. Inhibition of cysteine cathepsins had no effect, however, on collagen dissolution in the HaCaT II-4 model while serine proteinase blockade effectively attenuated collagen degradation [32]. These data reinforce the critical role for active plasmin, and not cathepsins, in the initiation of collagen degradation by TGF-*β*1 + EGF and are consistent with observations regarding the downregulation of several cathepsins by TGF-*β* [77].

The coupling of keratinocyte-based type-1 collagen degradation with plasminogen activation implicates intermediates other than MMP-10, including MMP-13 [78, 79]. The available evidence indicates the existence of a functional overlap between MMP-13 and uPA in the context of cutaneous wound repair, at least in the murine system [80]. Contrary to what has been observed in primary human keratinocytes, MMP-13 expression is, in fact, linked to transformation of human keratinocytes (i.e., HaCaT cells and various derivatives) [27, 74] and actually enhanced following addition of TGF-*β*1 to HaCaT II-4 cells [32, 74]. Use of a physiological 3-D reconstruct model of the stromal microenvironment, however, led to the conclusion that the combination of TGF-*β*1 + EGF does not significantly upregulate MMP-13 protein in HaCaT II-4 cells but, instead, leads to a robust induction of MMP-10 [32]. This disparity may be due in part to differences among the *ras*-HaCaT variants in MMP expression programs [81], TGF-*β*/EGF receptor crosstalk [11, 71, 82], or culture in 2D versus a more complex 3D stromal-equivalent system [32].

 Models of cutaneous injury repair have shed considerable light on the complex roles of growth factors and cascading protease systems in the tissue response to trauma. Generally, both TGF-*β*1 and EGF levels increase substantially following acute injury, partially due to their release from platelet *α* granules, but also through increased cellular expression, particularly at the wound edge [83]. These growth factors appear critical to the initial stages of cutaneous tissue regeneration through promotion of keratinocyte migration, as well as proliferation [84–87]. TGF-*β*1 and EGF upregulate MMP-10 in keratinocytes [72, 88] and, during cutaneous wound repair, MMP-10 is specifically localized to cells in the migrating tongue where it appears to enhance migration [87, 88]. Similarly, uPA and MMP-3, -9, and -13 all localize to leading edge epidermal cells [80]. Overexpression of constitutively active MMP-10 in the epidermis, moreover, has deleterious effects on the coordinated migration of keratinocytes into the wound bed; an effect attributed to excessive laminin-5 (laminin-332) processing [87]. Unconstrained MMP-10 activity leads to excessive collagenolysis [32] which impacts negatively on cell migration and, ultimately, the restoration of tissue integrity. Notably, PAI-1 expression also increases in keratinocytes at the wound margin and is deposited into the migration tracks of these cells, suggesting that this SERPIN, as well, plays an integral role in regulating directional migration and wound closure [89–92]. Coordinate upregulation of proteolytic enzymes such as the MMPs, together with the upstream inhibitor of plasmin generation (i.e., PAI-1) by individual growth factors provides an exquisite mechanism for fine control of focal proteolysis to facilitate optimal cell motility in complex environments.

## 5. Conclusions

 Increases in epithelial MMP-10 expression, and its subsequent activation by catalytic-levels of plasmin, mobilize an MMP cascade creating a proteolytic axis that accelerates collagen degradation through “superactivation” of MMP-1 while enhancing stromal proteolysis largely by MMP-7, -8, -9, and -13. Upregulation of the serine protease inhibitor PAI-1, in tumor cells, in mesenchymal cells within the tumor microenvironment as well as by “wound-stimulated” epithelial cells, may subsequently shift this proteolytic balance to optimize creation of a migratory “scaffold.” The available data clearly implicate PAI-1 as a major upstream modulator of a uPA→plasmin-generating system that exerts fine control over the MMP-dependent pericellular proteolytic cascade. In this context, PAI-1 may “titrate” the extent and locale of collagen matrix remodeling to facilitate cellular invasion within the stromal compartment as part of the metastatic and tissue repair programs. Further clarification of the complexity of controls and the extent of interdependency of individual cascading “arms” in this highly interactive network of matrix proteases and protease inhibitors will be necessary for the rationale design of focused therapeutic approaches for the treatment of cancer, fibrotic disorders and chronic wounds. Assessments of MMP inhibitors in clinical trials is already ongoing. Indeed, the emergence of uPA and PAI-1 as significant level-of-evidence-1 prognostic markers of overall survival in breast cancer is well established [93]. The development of small molecule inhibitors of PAI-1 (i.e., tiplaxtinin or PAI-039) that effectively attenuate aortic remodeling in the context of vascular injury [94] suggest that targeting this SERPIN may have translational implications for treatment of chronic fibrotic and, perhaps, malignant disease.
